# Scorpion toxin BmK I directly activates Nav1.8 in primary sensory neurons to induce neuronal hyperexcitability in rats

**DOI:** 10.1007/s13238-015-0154-4

**Published:** 2015-04-24

**Authors:** Pin Ye, Yunlu Jiao, Zhenwei Li, Liming Hua, Jin Fu, Feng Jiang, Tong Liu, Yonghua Ji

**Affiliations:** Laboratory of Neuropharmacology and Neurotoxicology, Shanghai University, Shanghai, 200436 China; Jiangsu Key Laboratory of Translational Research and Therapy for Neuro-Psycho-Diseases and the Second Affiliated Hospital of Soochow University, Institute of Neuroscience, Soochow University, Suzhou, 215021 China

**Keywords:** voltage-gated sodium channel, Na_v_1.8, primary sensory neurons, BmK I

## Abstract

Voltage-gated sodium channels (VGSCs) in primary sensory neurons play a key role in transmitting pain signals to the central nervous system. BmK I, a site-3 sodium channel-specific toxin from scorpion *Buthus martensi Karsch*, induces pain behaviors in rats. However, the subtypes of VGSCs targeted by BmK I were not entirely clear. We therefore investigated the effects of BmK I on the current amplitude, gating and kinetic properties of Na_v_1.8, which is associated with neuronal hyperexcitability in DRG neurons. It was found that BmK I dose-dependently increased Na_v_1.8 current in small-sized (<25 μm) acutely dissociated DRG neurons, which correlated with its inhibition on both fast and slow inactivation. Moreover, voltage-dependent activation and steady-state inactivation curves of Na_v_1.8 were shifted in a hyperpolarized direction. Thus, BmK I reduced the threshold of neuronal excitability and increased action potential firing in DRG neurons. In conclusion, our data clearly demonstrated that BmK I modulated Na_v_1.8 remarkably, suggesting BmK I as a valuable probe for studying Na_v_1.8. And Nav1.8 is an important target related to BmK I-evoked pain.

## Introduction

Primary sensory neurons in dorsal root ganglia (DRG) play an essential role in transmitting pain signals by detecting noxious stimuli through their peripheral axons and sending them to the postsynaptic neurons in spinal cord via their central axons (McCleskey and Gold, 1999; Todd, 2010). Voltage-gated sodium channels (VGSCs) are critical for the generation of action potentials (APs) in these neurons. Dynamic changes in the expression, trafficking, and function of VGSCs substantially enhance the excitability of neurons and spontaneous action potential firing and thus contribute to the initiation and maintenance of pain hypersensitivity in neuropathic and inflammatory chronic pain conditions (Dib-Hajj et al., 2010).

Up to nine tissue- or development-specific VGSC subtypes (Na_v_1.1-1.9) were identified and functionally characterized. According to their pharmacological sensitivity to tetrodotoxin (TTX), VGSCs can be divided into two groups: TTX-sensitive (TTX-S) and TTX-resistant (TTX-R). Nav1.8 sodium channel produces TTX-R current with a high activation threshold and slow inactivation kinetics and contributes to the upstroke of action potentials in small diameter nociceptive DRG neurons (Gold, 1999; Renganathan et al., 2001). The ectopic firing and neuronal hyperexcitability around the injured site along the peripheral axon are largely attributed to the redistribution or up-regulation of Nav1.8 (Gold et al., 1996). And the TTX-R current was enhanced, which contribute to the enhancement of neuronal excitability and thus to the pain and hyperalgesia associated with the chronic compression of the dorsal root ganglion (CCD) (Tan et al., 2006). Knock-down Nav1.8 expression by antisense oligodeoxynucleotides (AS-ONDs) reverse complete Freund’s adjuvant (CFA)-induced heat and mechanical hypersensitivity (Joshi et al., 2006; Khasar et al., 1998), spinal cord injury-evoked persistent pain (Yang et al., 2014). Therefore, Nav1.8 plays an indispensible role in neuronal hyperexcitability and spontaneous ectopic discharges under physiological and pathological conditions.

Many ion channel-targeted peptides isolated from animal venoms have been proven their value to gain insight into the role of ion channel in neuronal excitability and their gating mechanisms. Receptor site-3 on VGSCs is targeted by peptide toxins from scorpions, sea anemones, and spiders for the inhibition of sodium currents inactivation (Bosmans and Tytgat, 2007). To date, very few animal toxins have been shown to be capable of reshaping Nav1.8 current. Spider toxins ProTx-I and ProTx-II from the *Thrixopelma pruriens* tarantula were the first to be characterized as potent inhibitors of Na_v_1.8 opening (Priest et al., 2007). The μO-conotoxin MrVIB from cone snail *Conus marmoreus* was shown to be a Nav1.8 blocker at nanomolar concentrations (Ekberg et al., 2006; Knapp et al., 2012). To our knowledge, so far there had not been reported scorpion toxins which modulate Na_v_1.8 current.

BmK I, a sodium channel receptor site 3-specific modulator, isolated from the venom of scorpion *Buthus martensi Karsch* (BmK), is considered to be the key contributor of extreme pain-evoked by scorpion envenomation (Bai et al., 2010; Ji et al., 1996). Our previous work demonstrated that BmK I was able to modulate multiple subtypes of VGSCs, which delayed TTX-S sodium channels inactivation and aberrant I_Na_ at suprathreshold potentials in small-sized DRG and hippocampus neurons (Bai et al., 2006a; Chen et al., 2005). However, it was unclear which subtypes of TTX-R VGSCs is affected. Given the crucial roles of Na_v_1.8 in pain transmission, in the present study, we therefore investigated the direct modulation effects of BmK I on TTX-R Nav1.8 current in acute dissociated small-sized nociceptive rat DRG neurons. Our results suggested that BmK I prominently modulate Nav1.8 current, provide direct evidence to reminder that BmK I could induce inflammatory pain-related behaviors through acting on sodium channels, at least partial Na_v_1.8.

## Results

### BmK I enhanced Nav1.8 currents in small-sized DRG neurons

**Figure 1:**
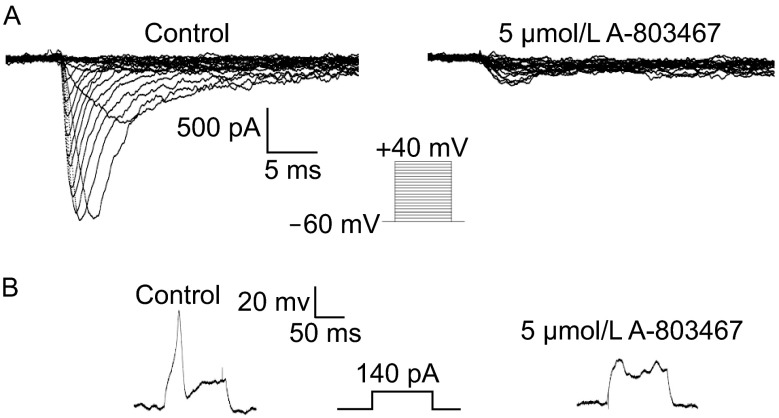
**Isolation of TTX-resistant Nav1.8 current and action potentials in DRG neurons**. (A) Representative Nav1.8 current was recorded from a small-sized DRG neuron using a voltage-protocol (as shown at middle) in the absence (left) or presence of A-803467 (5 μmol/L) (right). (B) Representative action potential was recorded from a small-sized DRG neuron by a current-protocol (as shown at middle) before and after application of A-803467 (5 μmol/L)

**Figure 2:**
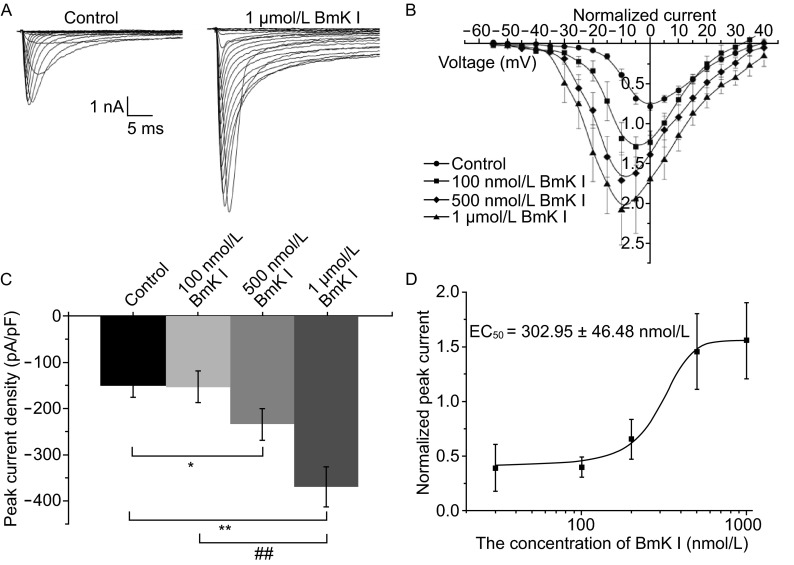
**BmK I increased Nav1.8 transient current in small DRG neurons**. (A) Representative Nav1.8 currents were recorded from a small-sized DRG neuron in the absence (left) and presence of 1 μmol/L BmK I. (B) I(V) relationship of Nav1.8 was determined before and after application of 100 nmol/L, 500 nmol/L or 1 μmol/L BmK I. Nav1.8 currents were normalized by respective maximum current under control condition. (C) Nav1.8 peak current densities were significantly enhanced in the presence of BmK I. (100 nmol/L: *n* = 7; 500 nmol/L: *n* = 6; 1 μmol/L: *n* = 10; control: *n* = 16; *P < 0.05, **P < 0.005, compared with control, respectively;^ ##^ *P* < 0.005, compared with 100 nmol/L). (D) The EC_50_ for BmK I effect determined by the increase of transient current in the presence of a series of BmK I concentration (30 nmol/L: 39 ± 21%, *n* = 5; 100 nmol/L: 40 ± 9%, *n* = 6; 200 nmol/L: 65 ± 18%, *n* = 5; 500 nmol/L: 146 ± 35%, *n* = 6; 1 μmol/L: 156 ± 35%, *n* = 7)

Nav1.8 current was elicited by 200 ms pulses depolarizing to +40 mV from a holding potential of −60 mV for inhibiting Nav1.9 currents (Fig. 1A). Nav1.8 current was isolated using TTX (500 nmol/L) in the bath solution and identified by Nav1.8 selective blocker A-803467 (5 μmol/L) (Fig. 1A). An action potential in the present of TTX of small-sized (diameter < 25 μm) DRG neurons was also blocked by application of A-803467 (Fig. 1B). Application of BmK I enhanced Nav1.8 current at all test potentials (Fig. 2A). I(V) relationship of Nav1.8 current, in control and series of BmK I concentration conditions, showed that BmK I have the potency on Nav1.8 current over the range from −30 mV to +40 mV (Fig. 2B). Nav1.8 current densities were increased significantly in the presence of BmK I (pA/pF, control: −150.5 ± 25.5; 100 nmol/L: −152.9 ± 34.5; 500 nmol/L: −235.4 ± 33.8; 1 μmol/L: −370.6 ± 44.1) (Fig. 2C). We found that the effect of BmK I on Nav1.8 transient current was dose dependent, whose EC_50_ value was 302.95 ± 46.48 nmol/L (Fig. 2D).

**Figure 3:**
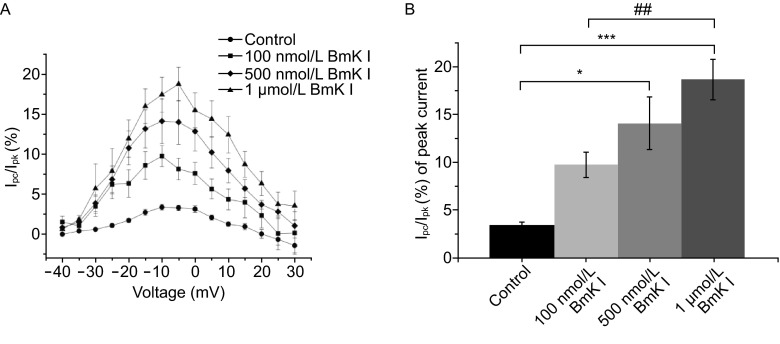
**BmK I increased Nav1.8 persistent currents**. (A) The percentage of persistent current over the voltage from −40 mV to +30 mV (the value of persistent current (I_pc_) divided by peak current (I_pk_)) in the absence and presence of BmK I. The values of persistent current were averaged by the data selected from the last 20 ms of Nav1.8 activation current. (B) I_pc_/I_pk_ at −5 mV were prominently enhanced under BmK I perfusion. (100 nmol/L: *n* = 6; 500 nmol/L: *n* = 6; 1 μmol/L: *n* = 10; control: *n* = 21; *P < 0.05, **P < 0.005, compared with control, respectively;^ ##^ *P* < 0.005, compared with 100 nmol/L)

The persistent current of Nav1.8 was evoked by depolarization for 200 ms, and measured by averaging the current amplitude recording of the last 10 ms for each pulse (Fig. 2A). The fraction of persistent current was prominent enhanced over the vast range of voltage (Fig. 3A). In maximal current, the fraction of persistent current was significantly enhanced in the presence of BmK I (%, control: 3.4 ± 0.4; 100 nmol/L: 9.8 ± 1.3; 500 nmol/L: 14.1 ± 2.7; 1 μmol/L: 18.7% ± 2.1) (Fig. 3B).

### BmK I shifted the kinetic curves of Nav1.8 in a hyperpolarizing direction

**Figure 4:**
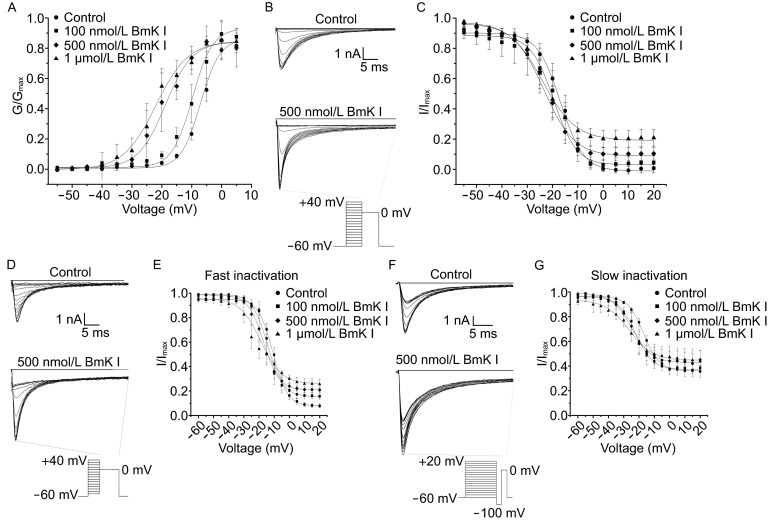
**BmK I shifted Nav1.8 kinetic curves of voltage-dependent activation, steady-state inactivation, fast and slow inactivation in a hyperpolarizing direction**. (A) The activation curves determined before and after application of BmK I (100 nmol/L, 500 nmol/L, 1 μmol/L). Nav1.8 activation currents were recorded as Fig. 1A and 1B, representative inactivation of Nav1.8 current traces in the absence and presence of BmK I (500 nmol/L). (C) Steady-state inactivation curves determined for control condition and in the presence of BmK I (100 nmol/L, 500 nmol/L, 1 μmol/L). (D and F) Representative fast and slow inactivation of Nav1.8 currents before and after application of BmK I (500 nmol/L). The currents were evoked by the voltage-protocol as shown at bottom. (E and G) Fast and slow inactivation curves were determined in the absence and presence of BmK I (100 nmol/L, 500 nmol/L, 1 μmol/L)

G(V) relationship indicated that BmK I shifted voltage-dependent activation of Nav1.8 in a hyperpolarizing direction (Fig. 4A). The midpoint of activation (V_1/2_) was significantly leftward shifted after BmK I treatment. The slope factor (*k*_m_) was not affected. Steady-state of Nav1.8 inactivation current was evoked by a 50 ms depolarizing pulse of 0 at the pre-pulse potentials ranging from −60 mV to +40 mV for 50 ms. Representative current traces in the absence and presence of 500 nmol/L BmK I were shown in Fig. 4B. The G(V) relationship showed that steady-state inactivation of Nav1.8 was also shifted by BmK I in a hyperpolarizing direction (Fig. 4C). The midpoint of inactivation (V_1/2_) was left shifted in the presence of 500 nmol/L and 1 μmol/L BmK I. Similar to the activation curves, the slope factor (*k*_m_) remained unchanged before and after application of BmK I.

Furthermore, we studied the voltage-dependence fast and slow inactivation by using protocols which with pre-pulse for different time duration (10 ms for fast inactivation and 200 ms for slow inactivation) under the impact of BmK I (Fig. 4). Representative current traces in control condition and presence of 500 nmol/L BmK I were shown in Fig. 4D. The G(V) relationship revealed that the fast inactivation of Nav1.8 was shifted in a hyperpolarizing direction (Fig. 4E). The midpoint of fast inactivation (V_f_) was left shifted in the presence of 500 nmol/L and 1 μmol/L BmK I, while the slope factors (*k*_f_) were not affected significantly. The slow inactivation of Nav1.8 was also affected by application of BmK I. Representative current traces were shown in Fig. 4F. G(V) curves showed BmK I also shifted leftward the slow inactivation of Nav1.8 (Fig. 4G). The midpoint (V_s_) was leftward shifted in the presence of 500 nmol/L and 1 μmol/L BmK I. In addition, 1 μmol/L BmK I increased the slop factor of slow inactivation (*k*_s_) to 7.67 ± 0.83 mV.

The detailed data were shown in Tables 1 and 2.

**Table 1:** **Activation and steady-state inactivation characteristics of Nav1.8 currents**

	Voltage-dependent activation			Steady-state inactivation		
	*n*	V_1/2_ (mV)	*k*_m_ (mV)	*n*	V_1/2_ (mV)	*k*_m_ (mV)
Control	19	−7.05 ± 2.40	3.25 ± 0.83	30	−16.89 ± 0.41	4.54 ± 0.28
100 nmol/L BmK I	10	−9.75 ± 2.31	3.39 ± 0.60	7	−19.52 ± 0.83	4.49 ± 0.51
500 nmol/L BmK I	5	−18.77 ± 2.66***	4.50 ± 0.83	5	−23.42 ± 0.90*	5.98 ± 0.60
1 μmol/L BmK I	6	−21.93 ± 1.25***	5.34 ± 0.51	7	−23.94 ± 0.68***	6.51 ± 0.45

Note: * * P* < 0.05, *** * P* < 0.001, compared with control group. Student’s *t*-test. Data are mean ± SEM

**Table 2:** **Fast and slow inactivation parameters of Nav1.8 currents**

	Fast inactivation			Slow inactivation		
	*n*	V_f_ (mV)	*k*_f_ (mV)	*n*	V_s_ (mV)	*k*_s_ (mV)
Control	25	−7.83 ± 0.29	4.76 ± 0.19	22	−16.97 ± 0.44	5.21 ± 0.26
100 nmol/L BmK I	4	−14.25 ± 0.73	5.11 ± 0.29	5	−22.35 ± 0.49	5.70 ± 0.30
500 nmol/L BmK I	6	−16.91 ± 0.55**	5.1 7 ± 0.25	5	−24.85 ± 0.70***	6.52 ± 0.45
1 μmol/L BmK I	7	−18.81 ± 1.04**	6.80 ± 0.66	8	−26.40 ± 0.90***	7.67 ± 0.83*

Note: * *P* < 0.05, ** P < 0.01, ***  *P* < 0.001, compared with control group. Student’s *t*-test. Data are mean ± SEM

### The effects of BmK I on Nav1.8 current was blocked by A-803467, a selective Nav1.8 blocker, in small-sized DRG neurons

**Figure 5:**
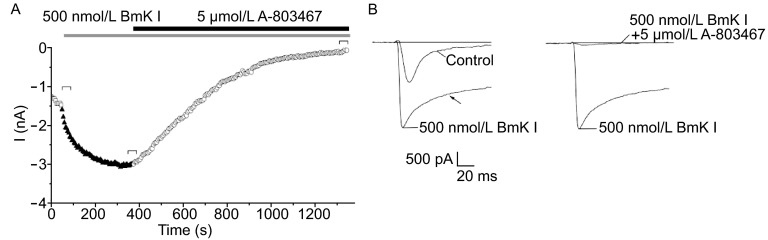
**Increase of Nav 1.8 currents caused by BmK I was blocked in the presence of A-803467 (5 μmol/L)**. (A) Increase of Nav 1.8 currents caused by BmK I was blocked in the presence of A-803467. Effects of BmK I (500 nmol/L) and A-803467 on Nav1.8 current recorded in a small-sized DRG neurons (13.72 pF). Nav1.8 current was elicited by depolarizing steps to 0 from a holding potential of −60 mV. (B) Current traces selected from (A) as indicated. The inactivation current trace was changed (arrow). And the current was almost completely blocked by A-803467. All DRG neurons for aforementioned experiments were exposed to 500 nmol/L TTX

Nav1.8 current was elicited by a 100-ms test pulse to 0 from a holding potential of −60 mV in a small-sized DRG neuron which exposed to the bath solution with 500 nmol/L TTX. The current was increased significantly in the presence of 500 nmol/L BmK I (Fig. 5A), and the effect was associated with the slowing in inactivation kinetics of current (Fig. 5B). After application of 5 μmol/L A-803467, the current was decreased and ultimately, almost completely disappeared (Fig. 5A and 5B), which suggesting that Nav1.8 was the target of BmK I, and the effect of BmK I on Nav1.8 can be abolished by Nav1.8 channel blocker.

### The excitability of small-sized DRG neurons enhanced by BmK I

**Figure 6:**
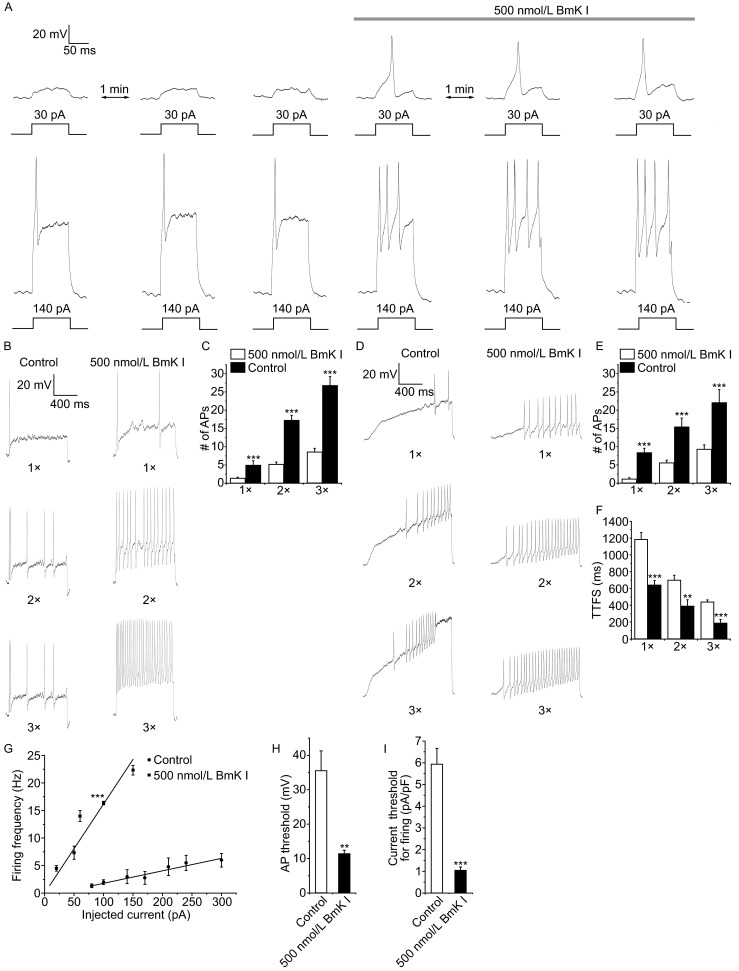
**BmK I lowered the threshold of excitability, increased firing that increased excitability in small DRG neurons**. (A) Current-clamp responses of a small-sized DRG neuron (17.32 pF) to 100 ms-low intensity (30 pA) or above threshold (140 pA) depolarizing pulses in the absence and presence of 500 nmol/L BmK I. Resting potential: −56 mV. (B) Representative traces of action potentials (Aps) induced by 1 s depolarizing current pulse at 1, 2 and 3 times rheobase in a small-sized DRG neuron (23.36 pF) under same conditions. Resting potential: −62 mV. (C) The average number of APs evoked by the current pulses was significantly increased in the presence of BmK I (1×: *n* = 5; 2×: *n* = 5; 3×: *n* = 6; control: *n* = 15, 9, 6; ***P < 0.001, compared with control, respectively). (D) Representative traces of APs induced by 1.5 s ramp current injection from 0 pA to 1, 2 and 3 times rheobase in a small-sized DRG neuron (23.36 pF) in the absence and presence of 500 nmol/L BmK I. Resting potential: −62 mV. (E) The average number of APs evoked by the ramp current pulses was significantly increased by BmK I (1×: *n* = 5; 2×: *n* = 6; 3×: *n* = 5; control: *n* = 12, 10, 9; ***P < 0.001; compared with control, respectively). (F) Time to the first spike (TTFS) was decreased by application of BmK I (1×: *n* = 5; 2×: *n* = 5; 3×: *n* = 4; control: *n* = 7, 8, 7; **P < 0.05, ***P < 0.001; compared with control, respectively). (G) Mean firing rate plotted divided by current intensity for DRG neurons in the absence and presence of BmK I (BmK I: *n* = 5; control: *n* = 7). Straight lines were linear fitted to data points, giving slope factors for control condition and BmK I perfusion (***P < 0.001). (H) Threshold of action potential in DRG neurons before and after BmK I treatment (BmK I: *n* = 6; control: *n* = 8; **P < 0.05). (I) Current of rheobase in DRG neurons in same conditions (BmK I: *n* = 8; control: *n* = 11; ***P < 0.001). Data normalized to the cell membrane capacitance

To determine whether the effects of BmK I on Nav1.8 current impact the excitability of DRG neurons, we employed current-clamp recording in small-sized (<25 μm) DRG neurons. Rheobase, action potential threshold, was the minimal injected current to evoke an action potential. Fig. 6A showed representative action potentials in response to low-intensity or rheobase current injection in the absence and presence of 500 nmol/L BmK I. Application of BmK I lowered the threshold of excitability and increased the number of action potentials in response to injected rheobase current.

Action potentials (APs) in response to rheobase (1×), 2 times (2×) and 3 times (3×) rheobase stimulation were recorded under the same conditions (Fig. 6B). Application of BmK I significantly increased numbers of APs evoked by all current stimulation (control: 1×, 1.4 ± 0.2; 2×, 5.1 ± 0.7; 3×, 8.5 ± 1.0; BmK I: 1×, 5. ± 1.2; 2×, 17.3 ± 1.3; 3×, 26.8 ± 2.4) (Fig. 6C). To further compare number of APs before and after BmK I treatment, we used 1.5 s ramp current pulse ranging from 0 pA to 1, 2 and 3 times rheobase (Fig. 6D). In the presence of 500 nmol/L BmK I, numbers of APs in responding to the test pulse were also significantly increased (control: 1×, 1.2 ± 0.3; 2×, 5.6 ± 0.8; 3×, 9.3 ± 1.2; BmK I: 1×, 8.4 ± 1.2; 2×, 15.5 ± 2.4; 3×, 22.2 ± 3.4) (Fig. 6E). In addition, the time to first spike (TTFS) in response to those current injection was significantly decreased after application of BmK I (ms, control: 1×, 1180.7 ± 85.0; 2×, 700.0 ± 56.0; 3×, 437.1 ± 27; BmK I: 1×, 644.0 ± 52.7; 2×, 394.0 ± 72.9; 3×, 192.5 ± 39.0) (Fig. 6F).

We next determined whether the firing of frequency was massively increased by BmK I. There was approximate 8 fold increase in the gain of frequency-stimulation relationship in DRG neurons (slopes of the frequency-current plots: BmK I, 0.16 ± 0.01; control, 0.02 ± 0.002) (Fig. 6G). The membrane potential appearing with first action potential was significantly reduced (mV, control: 35.5 ± 5.7; BmK I: 11.5 ± 0.9) (Fig. 6H). And the current required to reach firing threshold (rheobase) was also reduced significantly (pA/pF, control: 5.9 ± 0.7; BmK I: 1.1 ± 0.2) (Fig. 6I). These results indicated that BmK I enhances the excitability of small-sized DRG neurons.

## Discussion

Scorpion peptide toxins are documented to be valuable pharmacological tools or probes for studying the structure and function of the VGSCs (Bosmans and Tytgat, 2007). Our previous data showed that BmK I increased peak sodium currents and inhibited the inactivation of both TTX-S and TTX-R currents in small DRG neurons of rats (Bai et al., 2006b; Feng et al., 2008; Zhu et al., 2009), and effects of BmK I on TTX-S currents may be attributed to its modulation on Nav1.6, although possible effects on Nav1.7 could not be excluded (He et al., 2010; Zuo et al., 2006). Recent reports indicated that two distinctive components of sodium current, persistent current and resurgent current could be detected in DRG neurons of inflammatory models, among that the underlying mechanisms such as Nav1.8 was involved has been concerned (Tan et al., 2014). Our present data provided direct evidence to support that the effects of BmK I on TTX-R currents could be attributed to its effects on Nav1.8 sodium channel in DRG neurons. Our study indicated that BmK I delay the fast and slow inactivation of Nav1.8, that cause the deactivation of sodium channel uncompleted, thereby increase the persistent current, and then reduce the threshold of activation (Agrawal et al., 2001). It was firstly demonstrated that scorpion toxins, such as α-like toxin BmK I, could modulate Nav1.8 current remarkably, and reduced the threshold and increased the frequency of action potential mediated by Nav1.8. Thus, the direct modulation of Nav1.8 current by BmK I may contribute to the rapid enhanced excitability of primary sensory neurons and BmK I evoke-pain hypersensitivity in rats. However, the effect of BmK I on the Nav1.8 resurgent currents was also worthy of concern. It was speculated that the alternation of resurgent currents was closely related with the pain evoked by BmK I, which was the important next study.

The effect of BmK I on Nav1.8 persistent current was voltage dependence, which implied that the recognition and interaction between BmK I and Nav1.8 is associated with the voltage sensor of sodium channel. However, the possible domains are still unclear. Our and other’s results showed that the extracellular loop between S3 and S4 segments on DIV of sodium channels are critical for the binding of the receptor site 3 scorpion toxins (Bosmans and Tytgat, 2007). The Asp_1613_ in DIV S3-S4 loop seem to determine the interaction of classical α- and α-like toxins with sodium channels. The effects of α-toxin LqqV were decreased greatly due to mutation into Arg or His (Rogers et al., 1996). Similarly, substitution of Glu (homologous to E_1613_ in Nav1.2) contributed to BmK I-insensitive sodium channels (Nav1.1, Nav1.2, and Nav1.7) (Zuo and Ji, 2004). While, it was replaced by Ala in Nav1.8, which suggested this residue site is not necessary for BmK I acting on Nav1.8. It was speculated that the structure of the loop which would impair Nav1.8 interacting with BmK I may not be changed effectively by uncharged Ala with short-side chain. In addition, sequence analysis revealed that DIV S3-S4 linker is longer in Nav1.8 than in other sodium channels by four amino acids: Ser, Leu, Glu, and Asp (SLEN), which was considered that it is sufficient to make Nav1.8 resistant to α-toxin LqTx (Bosmans and Tytgat, 2007). However, our work showed that the sensitivity of Nav1.8 to BmK I is not deprived. Meanwhile, the insertion of two amino acid residues in S3-S4 linker in Nav1.5 (correspond to LE in Nav1.8) decrease the sensitivity to BmK I, but not deprive the effects of BmK I on Nav1.5 (unpublished data). This study suggested there are other potential precise mechanisms underlying the interaction between BmK I and Nav1.8. It warrants further investigation to test the effects of BmK I on Nav1.8 to improve our understanding of the biophysical properties and toxin pharmacology of sodium channels.

## Materials and methods

### Animals

All experiments followed European Community guidelines for the use of experimental animals and the policies issued by the International Association for the Study of Pain (Zimmermann, 1983). All animal experiments were performed with the approval of the Shanghai Animal Care and Use Committee. Adult male Sprague-Dawley rats weighing 80–120 g (purchased from Shanghai Experimental Animal Center, Chinese Academy of Sciences (CAS)).

### Drugs preparation and administration

The crude BmK venom was purchased from an individual scorpion culture farm in Henan Province, China. BmK I was purified according to previously described procedures (Ji et al., 1996).

### Whole-cell patch clamp recording on isolated DRG neurons of rats

Neurons were isolated from the DRG of adult rats according to aforementioned description (Chen et al., 2005). Briefly, the rats were anesthetized with ether and decapitated. Ganglia were dissected from the L3–L5 lumbar region. The trimmed ganglia were digested with collagenase type IV (2.67 mg/mL, Sigma, USA) and trypsin type I (1 mg/mL, Sigma, USA) at 37°C for about 30 min. Single cells were dissociated mechanically with a series of fire-polished Pasteur pipettes, plated on glass slides which covered with Poly-D-Lysine (PDL), then placed into dishes. The cells were cultured for 2 h in Dulbecco’s modified Eagle medium (DMEM F12; Gibco, Invitrogen, Grand Island, NY, USA) supplemented with 10% heat-inactivated fetal bovine serum (FBS; Gibco, Invitrogen). Culture dishes were incubated at 37°C in a humidified atmosphere containing 5% CO_2_.

Whole-cell path clamp recordings were performed on small-sized DRG neurons (diameter <25 μm). For voltage clamp recordings, the pipette solution contained 140 mmol/L CsCl, 1 mmol/L MgCl_2_, 10 mmol/L EGTA, 5 mmol/L Na_2_ATP, 0.4 mmol/L Na_2_GTP and 10 mmol/L HEPES, pH 7.2 (osmolarity, 300). The external solution contained 0.0005 mmol/L TTX, 140 mmol/L NaCl, 3 mmol/L KCl, 1 mmol/L MgCl_2,_ 1 mmol/L CaCl_2_, 0.1 mmol/L CdCl_2_, 10 mmol/L D-Glucose and 10 mmol/L HEPES, pH 7.3 (osmolarity, 320). For current clamp recordings, pipette solution contained 140 mmol/L KCl, 10 mmol/L NaCl, 0.5 mmol/L CaCl_2_, 1 mmol/L MgCl_2_, 10 mmol/L EGTA, 3 mmol/L Na_2_ATP, and 10 mmol/L HEPES, pH 7.2 (osmolarity, 300). The external solution contained 0.0005 mmol/L TTX, 150 mmol/L NaCl, 5 mmol/L KCl, 1 mmol/L MgCl_2,_ 2.5 mmol/L CaCl_2_,10 mmol/L D-Glucose and 10 mmol/L HEPES, pH 7.4 (osmolarity, 320). Both external solutions were saturated with O_2_.

Whole-cell path clamping experiments were performed by using an EPC-10 amplifier (HEKA eletronik, Germany) at room temperature. Patch pipettes were fabricated from glass capillary tubes by PP-830 Puller (Narishige, Japan) with the resistance of 3–5 MΩ. Data acquisition and stimulation protocols were controlled by Pulse/Pusle Fit 10.0 software (HEKA Elektronik). Capacitance transients and series resistance errors were compensated by 80%. Cells were discarded when the series resistance values were over 20 MΩ. Linear leakage was subtracted using P/4 protocol. Data were sampled at 50 kHz and low-pass filtered at 10 kHz.

### Electrophysiological protocols and data analysis

Nav1.8 currents and Nav1.8-meditated action potentials were isolated using TTX (500 nmol/L) in the bath solution and identified by Nav1.8 selective blocker A-803467 (5 μmol/L).

Mean conductance (G) was calculated from peak current (I)–voltage (V) relationships using the equation G = I/(V − V_r_), where I is the peak current elicited upon depolarization, V is the membrane potential, and V_r_ is the reversal potential.

Nav1.8 currents were elicited by 200 ms pulses depolarizing to +40 mV from a holding potential of −60 mV for inhibiting Nav1.9 currents. The voltage-dependent activation was fitted with the Boltzmann relationship, G/G_max_= 1/[1 + exp(V − V_1/2_)/*k*_m_], where V_1/2_ is the voltage for half-maximum activation and *k*_m_ is the slope factor.

Steady-state inactivation were evoked by a 50 ms depolarizing pulse of 0 at the pre-pulse potentials ranging from −60 mV to +40 mV for 50 ms. The voltage-dependent fast inactivation and slow inactivation were analyzed by using protocols which with pre-pulse for different time duration (10 ms for fast inactivation and 200 ms for slow inactivation), to potentials ranging from −60 mV to +40 mV with the increments of 5 mV followed by a test pules of 0 for 50 ms. Data were fitted to the two-state Boltzmann equation, I/I_max_ = 1/[1 + exp(V − V_1/2_)/*k*], where V is the membrane potential of the conditioning step, V_1/2_ is the membrane potential at which half-maximal inactivation is achieved, and *k* is the slope factor. The parameters for fast inactivation were characterized by the half-maximal voltage V_f_ and the slope factor *k*_f_; correspondingly V_s_ and *k*_s_ for slow inactivation.

The equation used for fitting the dose-response relationship was: I_norm_= A/[1 + ([BmK I]/EC_50_)^p^ ] + C, where I_norm_ is the measured and normalized peak current, E_50_ is the half maximal effective concentration, and p is the slop factor.

Data were analyzed using Origin 8.5 (OriginLab, USA), and they were presented as means ± SEM. The number of cells examined was represented by *n*. Student’s paired or unpaired *t*-tests were used for comparisons.

## Author contributions

Y.P., L.T. and J.Y.H. conceived and designed the experiments. Y.P., J.Y.L. and L.Z.W performed the experiments. Y.P. and J.F. analyzed the data. Y.P., J.Y.L, L.Z.W, H.L.M. and F.J. contributed reagents/materials/analysis tools. Y.P., L.T. and J.Y.H wrote the paper. All authors have reviewed the manuscript.

## Abbreviations

Aps: action potentials
BmK: *Buthus martensi Karsch*
DRG: dorsal root ganglia
TTX: tetrodotoxin
TTX-R: TTX-resistant
TTX-S: TTX-sensitive
VGSCs: voltage-gated sodium channels
